# Both aldosterone and spironolactone can modulate the intracellular ACE/ANG II/AT1 and ACE2/ANG (1‐7)/MAS receptor axes in human mesangial cells

**DOI:** 10.14814/phy2.14105

**Published:** 2019-06-04

**Authors:** Danielle Stoll, Rodrigo Yokota, Danielle Sanches Aragão, Dulce E. Casarini

**Affiliations:** ^1^ Escola Paulista de Medicina – Department of Medicine Nephrology Division Universidade Federal de São Paulo UNIFESP São Paulo Brazil

**Keywords:** Aldosterone, mesangial cells, renin‐ANG‐aldosterone system, spironolactone

## Abstract

The kidney is an important target of the renin‐ANG‐aldosterone system (RAAS). To date, several studies have demonstrated the existence of a local RAAS in various tissues, including the renal tissue. The mineralocorticoid aldosterone is known to play a critical role in the classical RAAS; however, its effect on mesangial cells (MCs) remains to be elucidated. Based on this, our aim was to investigate whether aldosterone stimulation can modulate the intracellular RAAS of immortalized human MCs by evaluating ANG‐converting enzyme (ACE)/ANG II/ANG II receptor type 1 (AT1) and ANG‐converting enzyme 2 (ACE2)/ANG (1‐7)/MAS receptor axes. To realise this, protein expression, enzyme activity, and immunofluorescence were performed under aldosterone stimulation and in the presence of the mineralocorticoid receptor (MR) antagonist spironolactone (SPI). We observed that high doses of aldosterone increase ACE activity. The effect of aldosterone on the catalytic activity of ACE was completely abolished with the pretreatment of SPI suggesting that the aldosterone‐induced cell injuries through ANG II release were attenuated. Aldosterone treatment also decreased the expression of MAS receptor, but did not alter the expression or the catalytic activity of ACE 2 and ANG (1‐7) levels. Spironolactone modulated the localization of ANG II and AT1 receptor and decreased ANG (1‐7) and MAS receptor levels. Our data suggest that both aldosterone and the MR receptor antagonist can modulate both of these axes and that spironolactone can protect MCs from the damage induced by aldosterone.

## Introduction

Since 1898, when Tigerstedt and Bergman described renin for the first time (Tigerstedt and Bergman 1898), numerous studies have examined the physiological and pathological roles of the renin‐ANG‐aldosterone system (RAAS) (Ghazi and Drawz 2017). Several reports have demonstrated the existence in various tissues of a local RAAS that exerts autocrine, paracrine, and/or intracrine effects that may resemble or differ from those of the circulating RAAS (Leung 2004; Durvasula and Shankland 2008; Liu and Liu 2017). Aldosterone is a mineralocorticoid critical to the classical RAAS, contributing to the control of the acid/base and sodium/potassium balance and affecting several tissues; however, its effect on the local RAAS of mesangial cells (MCs) has not yet been described (Quinn and Williams 1988; Spat and Hunyady 2004).

MCs were identified for the first time in 1933 by Zimmermann (1933) and are essential for maintaining the integrity of the glomerular microvascular bed and mesangial matrix homeostasis, while also being involved in the modulation of glomerular filtration. The embryological and morphological origin of MCs are the same as that of smooth muscle cells, with microfilaments associated with α‐actin, myosin, and tropomyosin in its cytoskeleton. Due to these features, MCs are able to contract and/or expand. Also, these cells are similar to macrophages and endothelial cells and may play phagocytic and mesangial matrix production and reabsorption activities, secreting a wide variety of hormones and cytokines (Stockand and Sansom 1998; Abboud 2012).

Recent studies show that high glucose concentrations stimulate local RAAS from different cells, including mesangial cells (Vidotti et al. 2004).

Due to the importance of the activities of these cells, and their localization, any change in MCs state may cause pathological disorders, such as metabolic, immunological, and hemodynamic unbalance (Abboud 2012). MCs are also able to synthesize many RAAS components and our group has previously identified many of these components within MCs like ANG‐converting enzyme ACE and ACE 2, for instance (Casarini et al. 1997; Andrade et al. 1998; Aragao et al. 2011).

ACE is a peptidyl‐dipeptidase A that converts angiotensin I (ANG I) into angiotensin II (ANG II) which is found as a membrane‐bound enzyme and as a circulating molecule in several body fluids (Soubrier et al. 1988). ANG II is a potent vasoconstrictor and the RAAS main effector peptide which interacts with G‐protein‐coupled receptor AT 1. ANG II pathological activities are related to fibrotic processes, glomerular diseases, hypertension and diabetes (Wolf 1995; Mezzano et al. 2001; Fyhrquist and Saijonmaa 2008; Ruster and Wolf 2013).

ACE 2 is a membrane protein that shares homology with ACE (Donoghue et al. 2000). ACE 2 cleaves ANG II into ANG (1‐7) and its downregulation leads to excessive ANG II accumulation that causes albuminuria and glomerular damage in diabetic nephropathy (Ye et al. 2006). ANG (1‐7) is a biologically active heptapeptide found in several tissues, including kidney, and in serum, which may act as a negative feedback hormone to the actions of ANG II (Rice et al. 2004; Shaltout et al. 2007). ANG (1‐7) interacts with G‐protein‐coupled membrane receptor MAS which is found in renal tissues (Santos et al. 2003; Alenina et al. 2008; Zimpelmann and Burns 2009).

In the present study, we focused on the activity and expression of the components of (ACE)/ANG II/ANG II receptor type 1 (AT1) and ACE2/ANG (1‐7)/MAS receptor axes under aldosterone stimulation. As aldosterone is involved in modulation of the classical RAAS (Quinn and Williams 1988; Bollag 2014) and changes to this system are associated with certain pathologies, such as kidney injury and hypertension (Burchill et al. 2008; Navar et al. 2011), we believe that evaluating the actions of aldosterone on these intracellular axes is essential to understand the activity of this local system in physiological and pathological states. Novel findings that may influence the treatment of different renal pathologies may result from such research.

## Material and Methods

### MC culture

Immortalized human MCs were purchased from Creative Bioarray (cat. No CSC‐7719W) and characterized according to morphological aspect of stellate cells. MCs were cultured in low‐glucose DMEM supplemented with 10% FBS, 2 g/L NaHCO_3_, 2.6 g/L HEPES, and 10,000 Ul/L penicillin in a CO_2_ incubator (containing 5% CO_2_ and 95% air) at 37°C.

### Experimental design (Aldosterone Stimulation)

Cultured MCs were divided into six groups and treated with different concentrations of aldosterone. Physiological groups received 1 nmol/L and 0.1 nmol/L according to literature (Wehling 1997; Maguire et al. 1999). Based on the physiological doses, we define 10‐fold higher (10 nmol/L) or lower (0.01 nmol/L) than the physiological concentration as our supra‐ and subphysiological doses. Control group received no aldosterone treatment; and spironolactone group was treated with 10 nmol/L spironolactone (SPI) for 1 h, before being exposed to 10 nmol/L aldosterone. In some experiments 100 nmol/L aldosterone treatment was also administered.

### Cell viability assay

MC viability was determined using the 3‐(4,5‐dimethylthiazol‐2‐yl)‐2,5‐diphenyltetrazolium bromide (MTT) assay (van de Loosdrecht et al. 1994). MCs were seeded in 96‐well plates and cultured for 2 days after which the medium was removed and replaced with fresh FBS‐free medium, along with aldosterone at different concentrations (as described above). The cells were then maintained at 37°C in an atmosphere containing 5% CO_2_ for 24 h (short stimulation) or 72 h (prolonged stimulation). Subsequently, serum‐free medium containing MTT (0.5 mg/mL) was added, followed 2 h later by isopropanol. Absorbance at 570 nm was measured with an Infinite 200 plate reader (TECAN, Männedorf, Switzerland).

### Western blotting analysis

MCs treated as described above were washed with ice‐cold PBS and lysed for 5 min on ice with lysis buffer (10 mmol/L Tris at pH 8.0, 150 mmol/L NaCl, 0.2 mmol/L sodium orthovanadate, 0.1% Triton X‐100, 0.5 mmol/L IGEPAL, and 1 protease inhibitor tablet, EDTA‐free from Thermo Fisher Scientific, Waltham, MA). The lysates were then cleared by centrifugation at 21,300*g* for 30 min at 4°C, and total protein was quantified with a Pierce^™^ BCA Protein Assay Kit (Thermo Fisher Scientific). Equal amounts of protein (15–20 μg) were separated on 10% polyacrylamide gels and electrotransferred to nitrocellulose membranes (Amersham Hybond ECL; GE Healthcare, Little Chalfont, UK) that were subsequently blocked for 1 h at room temperature in Tris‐buffered saline‐Tween (20 mM Tris‐base at pH 7.6, 150 mmol/L NaCl, and 0.1% Tween 20) containing 5% BSA. The membranes were then incubated with an anti‐ACE (H‐170, 1:500) or anti‐ACE2 (H‐175, 1:500) antibody (Santa Cruz Biotechnology, Dallas, TX) in blocking solution at 4°C overnight, before being washed and incubated with a diluted conjugated secondary antibody at room temperature for 1 h. Antibody binding was visualized using an enhanced chemiluminescence kit (Amersham ECL Select^™^; GE Healthcare), and chemiluminescent signals were quantified using ImageJ software.

### ACE activity assay

ACE catalytic activity was measured fluorometrically using 1 mmol/L *Z*‐Phe‐His‐Leu (Piquilloud et al. 1970; Friedland and Silverstein 1976) as a substrate. The standard assay buffer comprised of a solution of 100 mmol/L potassium phosphate (pH 8.3) containing 300 mmol/L NaCl and 10^−4^ M ZnSO_4_. Aliquots of the enzymes were incubated with 200 μL of assay solution, consisting of 1 mmol/L *Z*‐Phe‐His‐Leu in standard buffer, for 18 h at 37°C. The enzymatic reaction was halted by addition of 1.5 mL of 280 mmol/L NaOH. Ten minutes after addition of 100 μL of *o*‐phthaldialdehyde (20 mg/mL) in methanol, the fluorescent reaction was stopped using 200 μL of 3 N HCl. Levels of the product, l‐His‐Leu, were measured using an Infinite 200 fluorometer at excitation and emission wavelengths of 365 nm and 495 nm, respectively.

### ACE2 activity assay

ACE2 activity was measured fluorometrically using 10 μmol/L Mca‐APK(Dnp) (AminoTech, Diadema, Brazil) as a substrate, in a buffer consisting of 75 mmol/L Tris‐HCl and 1 mol/L NaCl (pH 6.5) containing 10 μmol/L captopril and 0.5 μmol/L ZnCl_2_. Complete, Mini, EDTA‐free Protease Inhibitor Cocktail (Roche, Basel, Switzerland) was added according to the manufacturer's instructions, with or without 10 μmol/L DX600 (an ACE2 inhibitor; AminoTech) (Vickers et al. 2002; Pedersen et al. 2011). After 30 min at 37°C, measurements were taken using an Infinite 200 fluorometer at excitation and emission wavelengths of 320 nm and 420 nm, respectively.

### Immunofluorescence

MCs were cultured directly on sterilized coverslips, fixed with 4% paraformaldehyde in PBS, washed for 10 min in 0.1 mol/L glycine, and permeabilized for 10 min in PBS containing 0.2% Triton X‐100. The fixed cells were then incubated for 30 min with blocking buffer (5% albumin in PBS), followed by an anti‐ACE (H‐170), anti‐ACE2 (H‐175), anti‐MAS (H‐117), or anti‐AT1 (N10) antibody obtained from Santa Cruz Biotechnology, or an anti‐ANG (1‐7) or anti‐ANG II antibody kindly donated by Dr. Preenie Senanayake of the Cole Eye Institute, Cleveland, Ohio, USA. The cells were subsequently exposed to an Alexa Fluor^®^ 594‐conjugated antirabbit IgG antibody for 1 h, and mounted in VECTASHIELD^®^ Mounting Medium with DAPI (Vector Laboratories, Burlingame, CA). Images were captured with an AxioCam MRc microscope camera, with magnification of 100× in Rhod filter (Zeiss, Oberkochen, Germany) and analyzed using ImageJ software. Results were expressed as mean fluorescence intensity.

### Statistical analysis

Statistical analysis was performed using GraphPad Prism Version 5 (GraphPad Software, La Jolla, CA). Statistically significant differences were determined by one‐way ANOVA followed by Tukey's multiple comparison test. where *P* value is described as * (0.01‐0.05), ** (0.001‐0.01) and *** (< 0.001). Results are reported as mean with SEM, *n* represents the number of wells and each experiment was repeated a minimum three times.

### Ethical statement

This project was approved by the Research Ethics Committee of the Federal University of São Paulo (approval number 6616050315).

## Results

### Prolonged exposure to physiological doses of aldosterone improved MC viability

**Figure 1:**
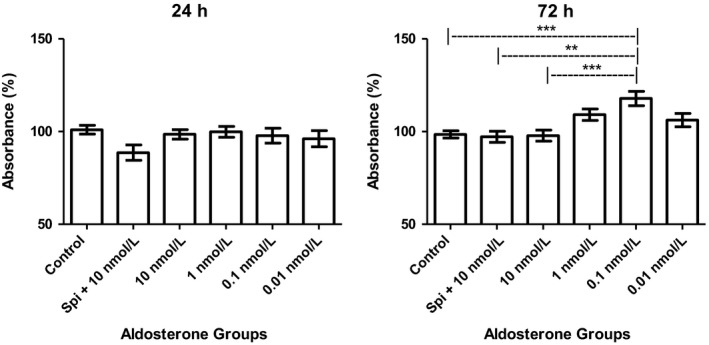
MTT viability assay performed after 24 h or 72 h of aldosterone stimulation (*n *= 4, SPI group *n *= 3, ANOVA *P *< 0.0001), Tukey's test **indicates value of *P* = 0.001–0.01, ***indicates value of *P* < 0.001.

MCs treated with a physiological dose of aldosterone (0.1 nmol/L) for a prolonged period (72 h) exhibited significant increases in viability compared with the control group (19.37%), SPI group (20.65%), and 10 nmol/L group (20.05%), as shown in Figure 1. No differences in viability were noted between the groups after the shorter (24 h) exposure time.

### ACE expression was not altered by aldosterone treatment

**Figure 2:**
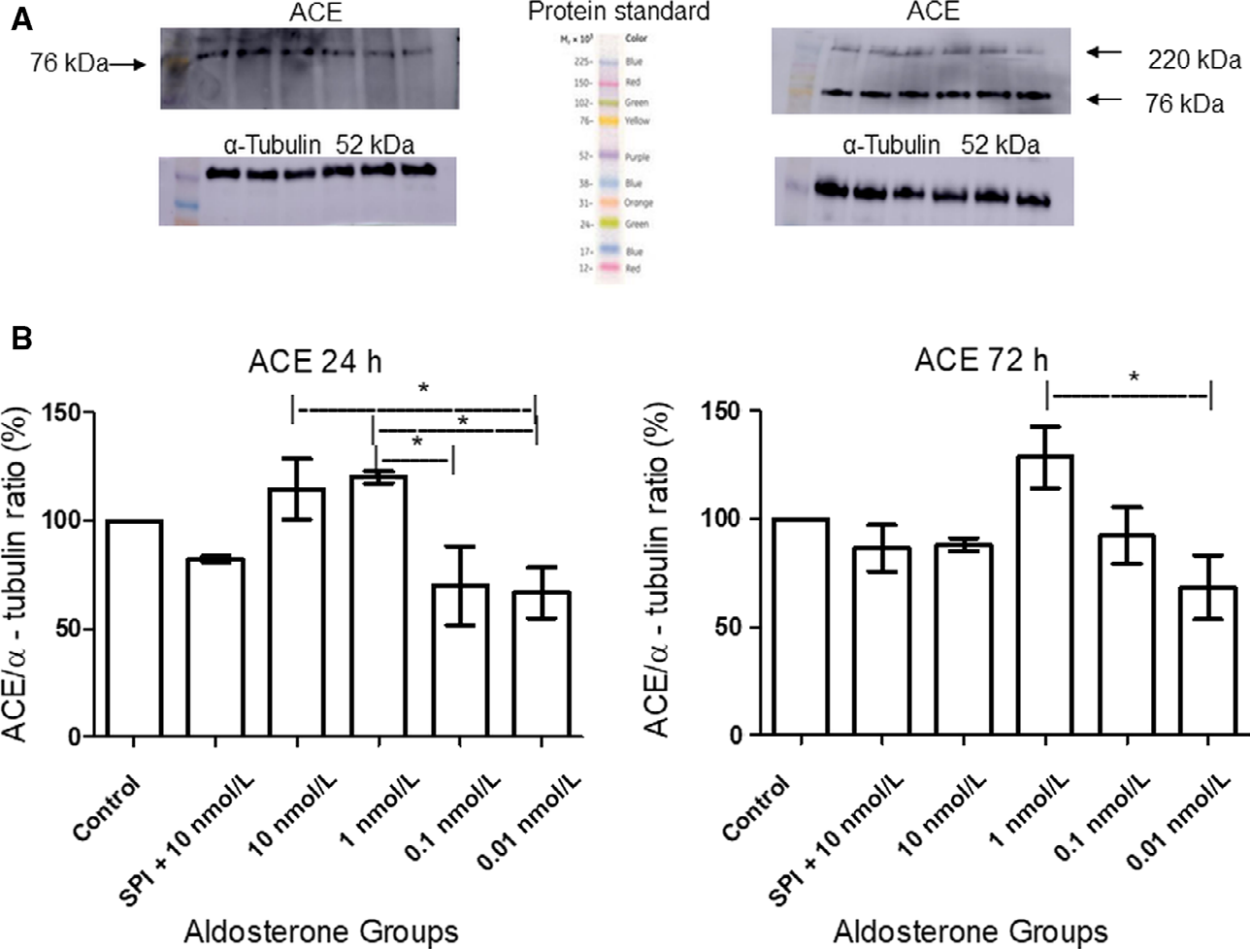
Western blotting analysis of ACE expression in MCs after 24 h (*n* = 3, ANOVA *P* = 0.01; Tukey's test *indicates value of *P* = 0.01–0.05) and 72 h (*n *= 3, ANOVA *P* = 0.01; Tukey's test *indicates value of *P* = 0.01–0.05) of aldosterone stimulation. (A) The bands of approximately 76 kDa and 220 kDa (the latter was not analyzed) correspond to the low and high molecular weight ACE isoforms, respectively. The α‐tubulin band (52 kDa) was used as an endogenous control. (B) Quantitative analysis of the relative intensities of the bands.

Compared with the control group, aldosterone administration did not induce changes in ACE expression. However, significant differences were noted between the high‐ and low‐dose groups (Fig. 2). After 24 h of exposure, ACE expression was 48.08% higher in the 10 nmol/L than the 0.01 nmol/L group, 50.03% higher in the 1 nmol/L than the 0.1 nmol/L group, and 53.47% higher in the 1 nmol/L than the 0.01 nmol/L group. After 72 h of aldosterone stimulation, the differences between these groups were smaller, with only the 1 nmol/L and 0.01 nmol/L groups significantly differing in this respect (ACE levels were 60.17% higher in the former than the latter).

### SPI decreased ACE levels in MCs

**Figure 3:**
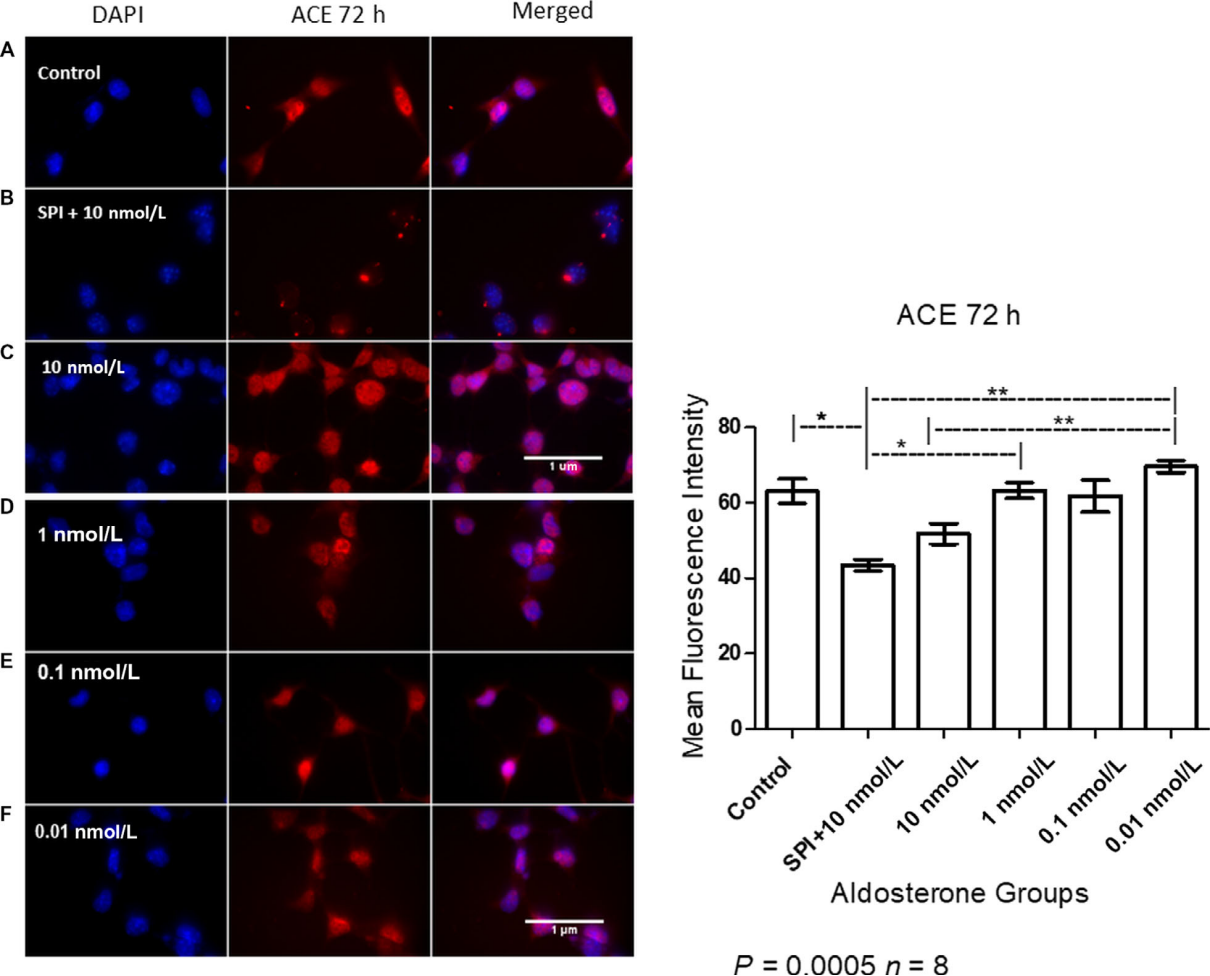
Immunofluorescent staining of ACE in MCs after 72 h of stimulation with aldosterone at different concentrations (A = Control, B = SPI+10 nmol/L, C = 10 nmol/L, D = 1 nmol/L, E = 0.1 nmol/L, F = 0.01 nmol/L of aldosterone). Nuclei are stained in blue and ACE in red. Original magnification: 100×. ANOVA *P* = 0.0005; Tukey's test *indicates value of *P* = 0.01–0.05, **indicates value of *P* = 0.001–0.01.

Staining with an anti‐ACE antibody revealed that ACE levels were significantly reduced (19.64%) in MCs incubated with both 10 nmol/L SPI and aldosterone in comparison to control group. Also, significant differences were found between the SPI, high‐ and low‐ dose groups. The physiological concentrations (1 and 0.1 nmol/L aldosterone treatment) exhibited ACE media levels very similar to the control group (63.25, 61.74 and 63.06 respectively) (Fig. 3).

### Aldosterone increased ACE catalytic activity

**Figure 4:**
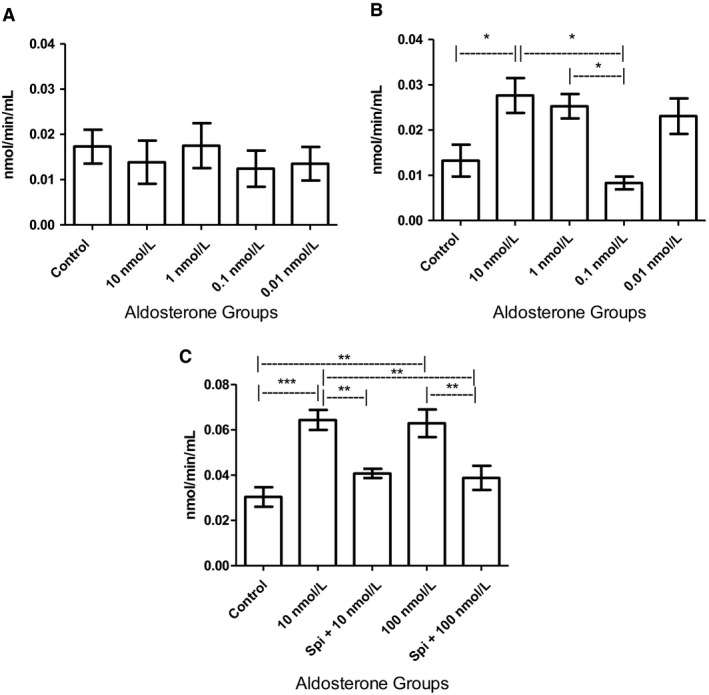
ACE catalytic activity. (A) After 24 h of aldosterone stimulation, no statistically significant differences were noted between the groups (*n* = 4). (B) After 72 h of stimulation, high doses of aldosterone were found to increase ACE catalytic activity (*n* = 3; ANOVA *P* = 0.003; Tukey's test *indicates value of *P* = 0.01–0.05.). (C) SPI treatment for 1 h attenuated the effect of aldosterone on ACE activity in MCs cultured for 72 h (*n* = 3; ANOVA *P* < 0.0001; Tukey's test **indicates value of *P* = 0.001–0.01, ***indicates value of *P* < 0.001.

After 72 h of stimulation, ACE catalytic activity in MCs treated with 10 nmol/L aldosterone was increased twofold compared to that in the control cells, as shown in Figure 4B. In addition, such activity in cells stimulated with 10 nmol/L aldosterone was threefold higher than that in cells treated with a physiological dose (0.1 nmol/L). SPI supplementation attenuated these effects, even at aldosterone concentrations up to 100 nmol/L (Fig. 4C). Compared to the control, ACE activity was not altered in MCs stimulated with aldosterone over a short period (24 h) (Fig. 4A).

### SPI resulted in localization of ANG II to the nuclei of MCs

**Figure 5:**
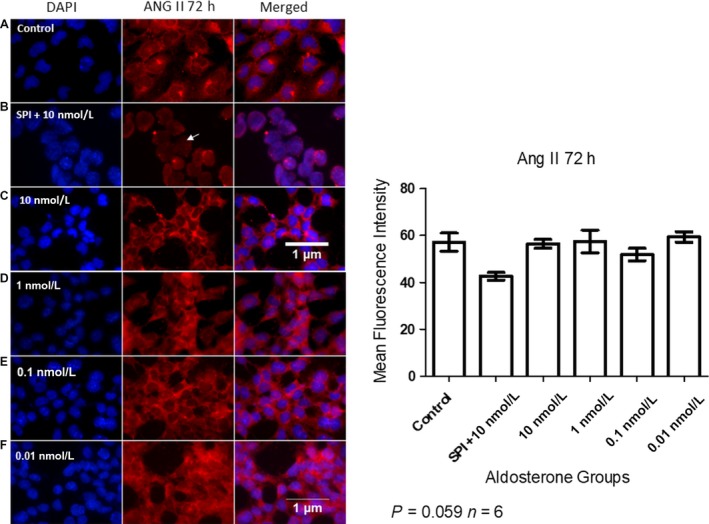
Immunofluorescent detection of ANG II localization in MCs after 72 h of aldosterone stimulation (A = Control, B = SPI+10 nmol/L, C = 10 nmol/L, D = 1 nmol/L, E = 0.1 nmol/L, F = 0.01 nmol/L of aldosterone). Nuclei are stained in blue and ANG II in red. The arrow indicates nuclear internalization of ANG II in MCs pretreated with SPI. Original magnification: 100×.

ANG II staining was more intense in the cytoplasm than the nuclei of MCs in the control group. MCs stimulated with different doses of aldosterone had higher levels of ANG II in the cytoplasm, with weak staining evident in the nucleus compared to those in the control group (Fig. 5). In MCs pretreated with 10 nmol/L SPI, ANG II was localized in the nucleus (arrow) and no fluorescence was detected in the cytoplasm.

### SPI treatment modulated AT1 receptor localization

**Figure 6:**
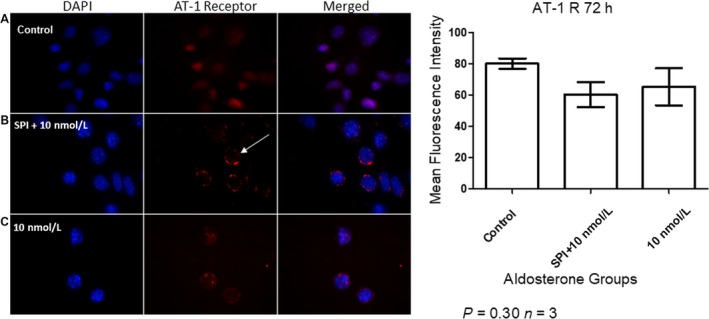
Immunofluorescent detection of AT1 receptor localization in MCs after 72 h of aldosterone stimulation. A = Control, B = SPI + 10 nmol/L, and C = 10 nmol/L. Nuclei are stained in blue and the AT1 receptor in red. Arrow indicates AT1 receptor presence on the nuclear membrane. Original magnification: 100×.

The AT1 receptor was localized within the nuclei of MCs in the control group, whereas it was found to be present in the nuclear membrane (arrow in Fig. 6) of those administered SPI prior to aldosterone. In cells stimulated with 10 nmol/L aldosterone, this receptor was observed in the nucleus and perinuclear region, as shown in Figure 6.

### Aldosterone modulated glycosylation of ACE2

**Figure 7:**
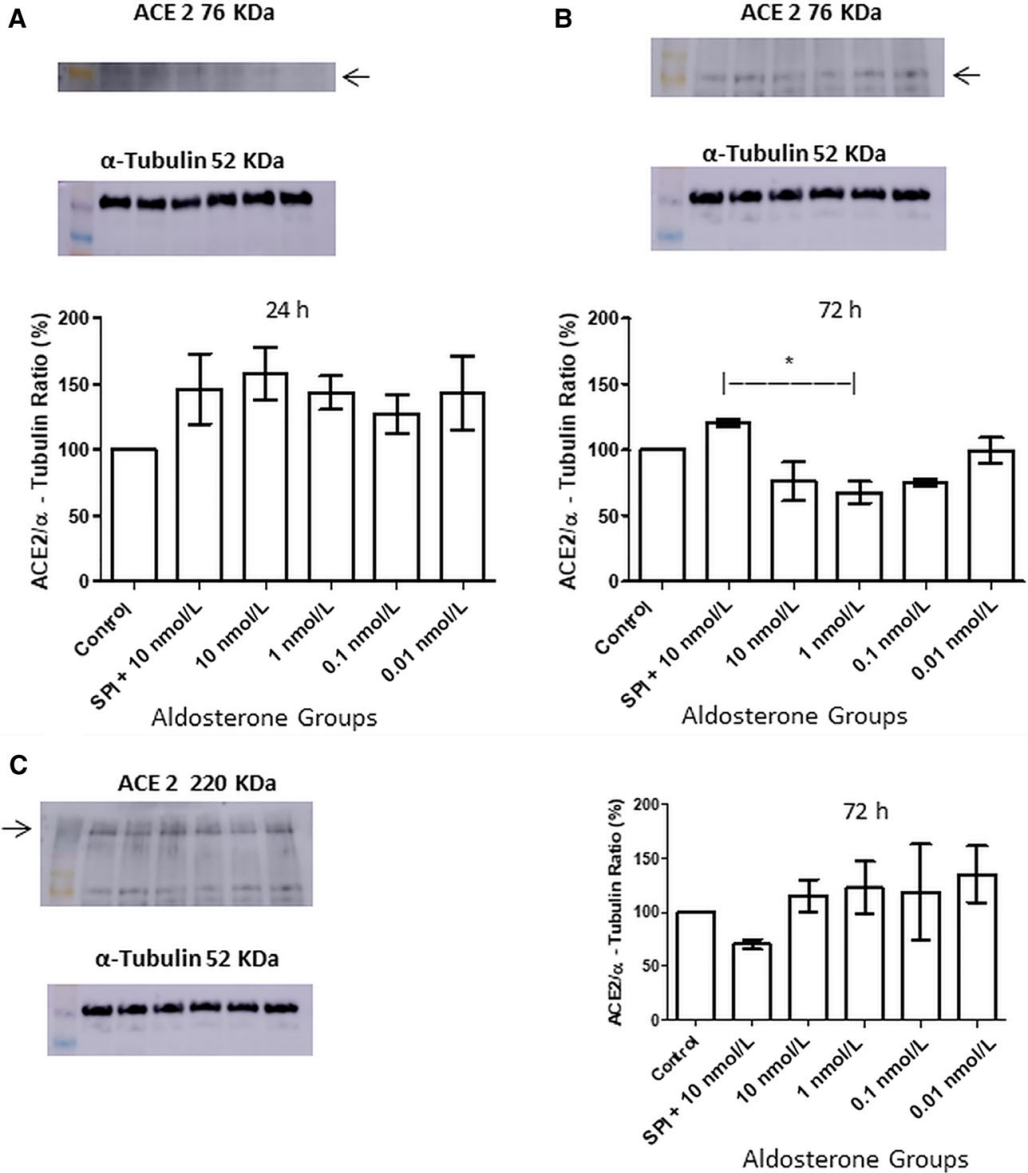
Western blotting analysis and quantification of nonglycosylated ACE2 (approximately 76 kDa) expression in MCs after (A) 24 h and (B) 72 h (*n *= 3, ANOVA *P* = 0.02; Tukey's test *indicates value of *P* = 0.01–0.05.) of aldosterone stimulation. (C) Glycosylated ACE2 (approximately 220 kDa) levels after 72 h of aldosterone stimulation.

Stimulation with aldosterone for 24 h did not alter ACE2 expression, as shown in Figure 7A. With respect to cells stimulated with aldosterone for 72 h, aldosterone receptor blockade with SPI for 1 h prior to 10 nmol/L aldosterone treatment induced a 52.29% significant increase in the level of nonglycosylated ACE2 compared to the 1 nmol/L aldosterone‐only group, (Fig. 7B). Glycosylated ACE2 levels were higher in the aldosterone‐treated groups and lower in the SPI‐pretreated group although this result was not statistically significant (Fig. 7C).

### Total ACE2 expression was not affected by aldosterone

**Figure 8:**
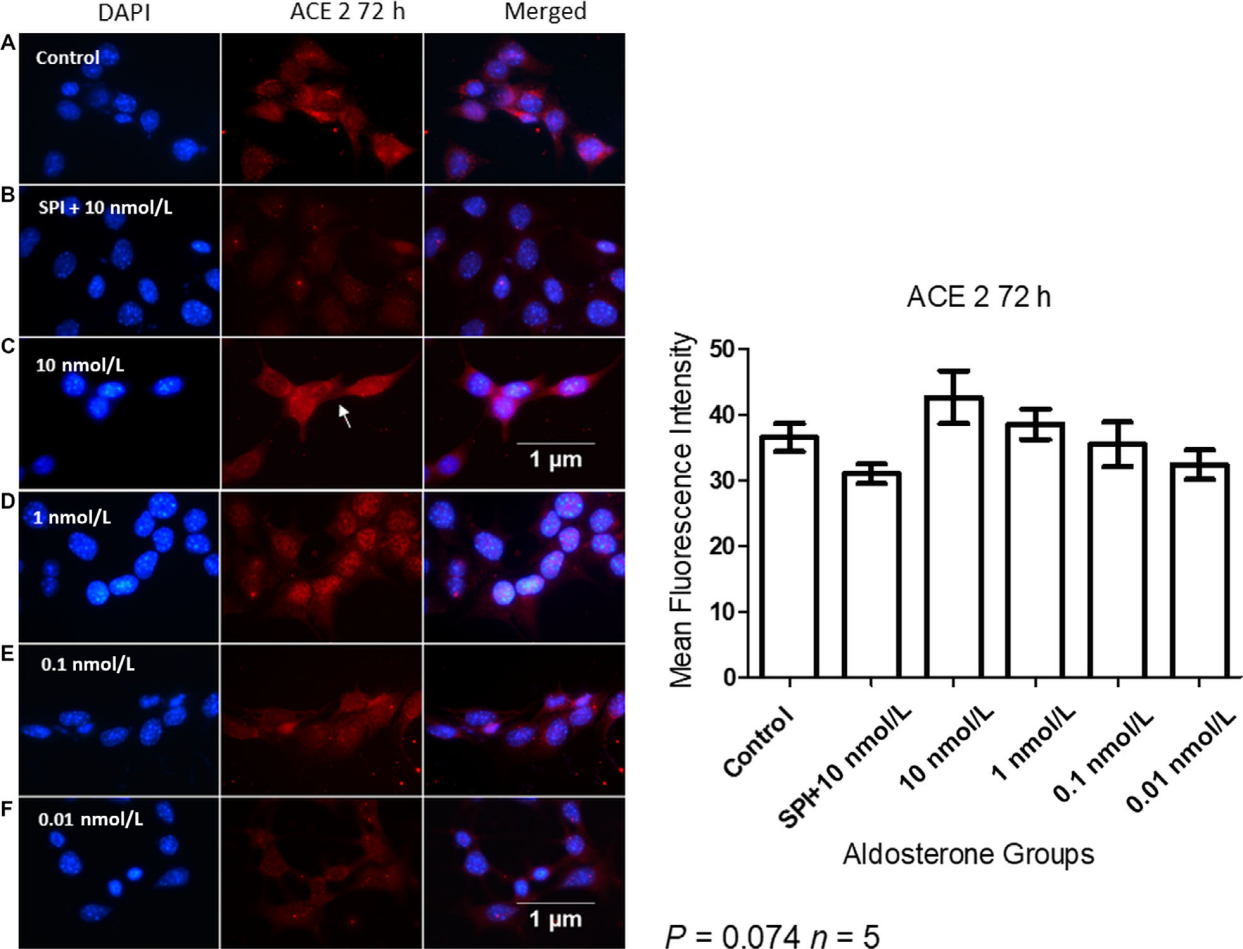
Immunofluorescent detection of ACE2 localization in MCs after 72 h of aldosterone stimulation (A = Control, B = SPI+10 nmol/L, C = 10 nmol/L, D = 1 nmol/L, E = 0.1 nmol/L, F = 0.01 nmol/L of aldosterone). Arrow indicates cytoplasmic ACE2 localization. Nuclei are stained in blue and ACE2 in red. Original magnification: 100×.

MCs pretreated with 10 nmol/L SPI exhibited a nonsignificant decrease in the expression of ACE2. Cells treated with 10 nmol/L aldosterone demonstrated an increase in cytoplasmic ACE2 localization (arrow in Fig. 8C). The physiological (1 nmol/L and 0.1 nmol/L) and 0.01 nmol/L doses resulted in an ACE2 staining pattern similar to that in the control group (Fig. 8).

### ACE2 activity was not affected by aldosterone

**Figure 9:**
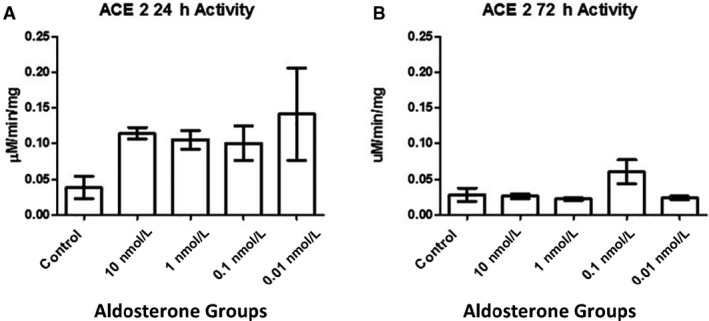
ACE2 catalytic activity, determined using Mca‐APK(Dnp) as a substrate. The assay was performed using sample sizes of *n* = 4 for (A) 24 h of aldosterone stimulation and *n* = 3 for (B) 72 h of stimulation (*P* > 0.05).

No change in ACE2 catalytic activity was noted in MCs following exposure to aldosterone (Fig. 9), regardless of exposure time.

### Spironolactone decreased ANG (1‐7) in MCs

**Figure 10:**
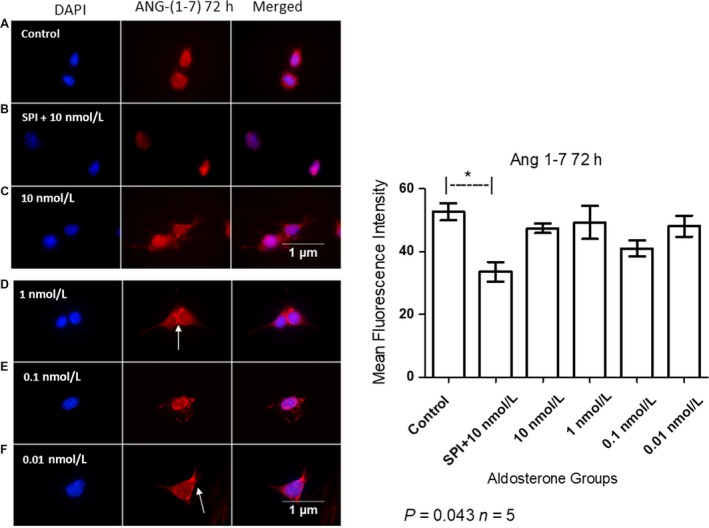
Immunofluorescent detection of ANG (1‐7) localization in MCs after 72 h of aldosterone stimulation (A = Control, B = SPI+10 nmol/L, C = 10 nmol/L, D = 1 nmol/L, E = 0.1 nmol/L, F = 0.01 nmol/L of aldosterone). Arrows indicate ANG (1‐7) concentration in the nuclear membrane. Nuclei are stained in blue and ANG (1‐7) in red. Original magnification: 100×. ANOVA *P* = 0.043; Tukey's test *indicates value of *P* = 0.01–0.05.

MCs pretreated with 10 nmol/L SPI exhibited a decrease in the expression of ANG (1‐7) approximately 20% in comparison to the control group and was localized in the nuclear region of these cells. Aldosterone groups demonstrated a similar pattern of cytoplasmic and nuclear ANG (1‐7) localization, whereas in those treated with 1 nmol/L and 0.01 nmol/L aldosterone, this peptide was concentrated in the nuclear membrane (arrows in Fig. 10). Control group exhibits ANG (1‐7) in nuclear and perinuclear region but not in the cytoplasm.

### Aldosterone and spironolactone decreased MAS receptor expression

**Figure 11:**
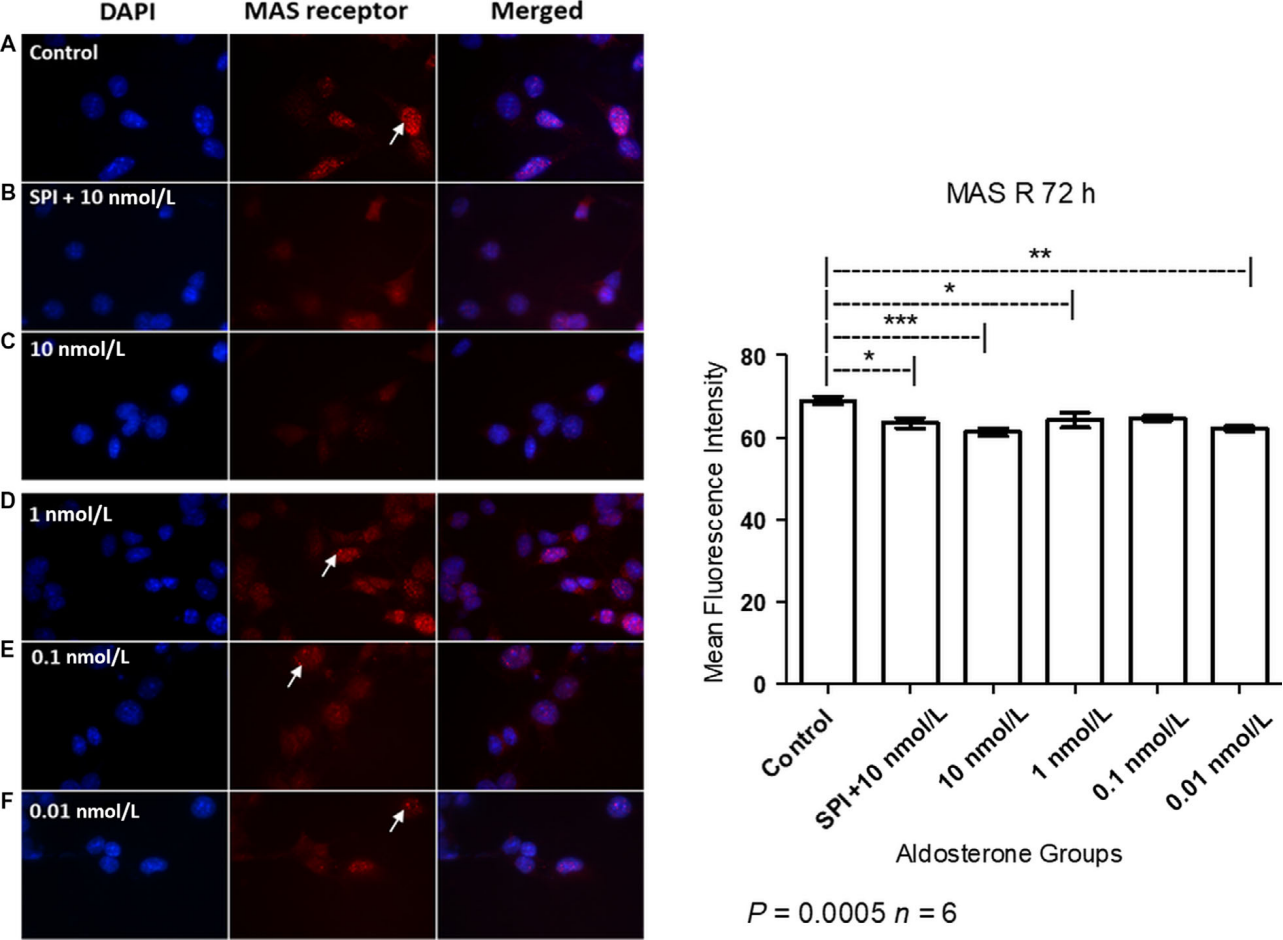
Immunofluorescent detection of MAS receptor localization in MCs after 72 h of aldosterone stimulation (A = Control, B = SPI+10 nmol/L, C = 10 nmol/L, D = 1 nmol/L, E = 0.1 nmol/L, F = 0.01 nmol/L of aldosterone). Arrows show a similar stain pattern of MAS receptor between control, physiological doses (1–0.1 nmol/L) and 0.01 nmol/L aldosterone treatment in nuclear region. Nuclei are stained in blue and the MAS receptor in red. Original magnification: 100×. ANOVA *P* = 0.0005; Tukey's test *indicates value of *P* = 0.01–0.05, **indicates value of *P* = 0.001–0.01, ***indicates value of *P* < 0.001.

MAS receptor expression was decreased in cells treated with aldosterone and spironolactone (Fig. 11). Besides the reduction in MAS receptor levels, images show a similar stain pattern between control, physiological doses (1–0.1 nmol/L) and 0.01 nmol/L aldosterone treatment in nuclear region (arrows).

## Discussion

Our data indicated that prolonged stimulation with a physiological dose (0.1 nmol/L) of aldosterone increased the viability of MCs by approximately 20% compared with the control group and 10 nmol/L aldosterone treatment (Fig. 1). Other cell types, such as endothelial cells and β‐cells, do not exhibit a decrease in their viability when low doses of aldosterone are applied (Chen et al. 2015; Zhang et al. 2016). Studies show a crosstalk between MCs, podocytes and glomerular endothelial cells suggesting that damage to one of these cells may lead to injuries in other cell types (Leveen et al. 1994). Our data suggest that physiological concentrations of aldosterone are important to the health of MCs and that an increase in their viability may provide a good environment not only for MCs, but also for the surrounding cell types. Western blotting showed that after 24 h of aldosterone stimulation, the highest doses resulted in greater expression of ACE with a mass of approximately 76 kDa than the lowest doses. However, the aldosterone treatment groups did not significantly differ from the control, suggesting that aldosterone does not affect local ACE production and that there may be a range of ACE levels associated with high and low aldosterone doses that are not deleterious to MCs.

Following immunostaining, MCs pretreated with SPI exhibited weaker ACE signals (Fig. 3) than those in any of the other groups, indicating that SPI treatment can heavily decrease ACE levels, in contrast to our western blotting results (Fig. 2). This discrepancy may be explained by the fact that in our immunofluorescence experiment, both the high and lowmolecular‐weight isoforms were stained in whole cells, whereas our western blotting protocol only included quantification of the latter, and only in lysates. Despite this, we observed that prolonged exposure to aldosterone at high doses induced increased ACE catalytic activity, and SPI pretreatment entirely negated these effects, even at aldosterone concentrations of up to 100 nmol/L (Fig. 4).

Our data also showed that ANG II levels were predominantly increased in the cytoplasm following administration of 10 nmol/L aldosterone; however, when cells were treated beforehand with 10 nmol/L SPI, ANG II was only observed in the nuclei, indicating that it is translocated to the nucleus in the presence of this mineralocorticoid receptor (MR) antagonist (Fig. 5). Nuclear accumulation of ANG II has also been noted in other tissues and cells, including the brain, liver, and endothelial cells (Booz et al. 1992; Erdmann et al. 1996). Tadevosyan et al. (Tadevosyan et al. 2010, 2012) demonstrated that exposure of isolated cardiac cell nuclei to ANG II induces inositol 1,4,5‐trisphosphate‐dependent Ca^2+^ release and de novo RNA synthesis. They also showed that classical ANG receptor blockers and ACE inhibitors fail to prevent the intracrine actions as described for ANG II. This suggests an important role for this peptide that warrants further study to elucidate its function within the nuclei of MCs.

Identification of the pathway with which SPI interacts to induce nuclear internalization of ANG II is also necessary. Our own data revealed that AT1 receptor is affected in MCs pretreated with SPI (Fig. 6). Shelat et al. (1999 ) suggested that aldosterone modulates AT1 receptor activation by binding the glucocorticoid receptor, and not the MR. However, our data demonstrated that in the presence of SPI, the AT1 receptor was localized to the nuclear membrane, while ANG II was found within the nucleus. In addition, 10 nmol/L aldosterone treatment resulted in the presence of AT1 receptor within both the nucleus and perinuclear membrane, with ANG II being observed in the cytoplasm and perinuclear membrane. This suggests that ANG II does not bind the AT1 receptor in the presence of MR blockers. These discrepant results may be due to the different aldosterone doses and MR blocker types used. Moreover, SPI is known to be nonspecific, being also able to bind the GR receptor (Garthwaite and McMahon 2004), which might explain the modulation of AT1 receptor activation observed here.

Glycosylation of ACE2 was altered by aldosterone and its antagonist (Fig. 7). Baudin and colleagues (Baudin et al. 1997) have indicated that N‐glycosylation of somatic ACE appears to have a role in its intracellular targeting and protection against hepatic lectins, as well as expression of the membrane‐bound form and secretion of the soluble form. However, to the best of our knowledge, no study has yet described the effects of ACE2 glycosylation.

Although our data demonstrated a nonsignificant decrease in ACE2 levels after SPI administration, in all other groups ACE2 was found in a more similar profile in comparison to the control group than the pattern found in cells treated with SPI. (Fig. 8).

Takeda et al. (2007) have shown that in Dahl salt‐sensitive rats fed a high‐sodium diet, the ANG II receptor antagonist candesartan increases cardiac ACE2 protein expression, whereas the aldosterone receptor antagonist eplerenone does not. In addition, in Dahl salt‐resistant rats, neither of these drugs alters ACE2 protein levels in cardiac tissue. Karram et al. (2005) obtained different results, showing that administration of SPI to rats with cardiac hypertrophy increases expression of ACE2 and decreases that of ACE. However, the pathological roles of ACE2 remain to be elucidated, since studies have shown increased *ACE2* mRNA expression in patients with ischemic and idiopathic cardiomyopathy and elevated ANG (1‐7) levels in subjects with cardiac insufficiency, which according to these authors may reflect either the deleterious effects of excessive ACE2 activity or a physiological attempt to protect cardiac tissue through the ACE2/ANG (1‐7)/MAS receptor axis (Zisman et al. 2003; Goulter et al. 2004). Notably, Crackower et al. (2002) showed that ACE2‐knockout mice exhibit a severe dysfunction in myocardial contractility, in association with increased ANG II levels.

Our data showed that ACE2 catalytic activity was not altered by aldosterone stimulation (Fig. 9). However, localization of ANG (1‐7) was affected by aldosterone treatment, as shown in Figure 10. Although MCs pretreated with SPI exhibited a non‐significant reduction in ACE2 protein levels, ANG (1‐7) was decreased in this group. Zimpelmann and Burns showed that in human mesangial cells, ANG (1‐7) stimulates the phosphorylation of MAPKs and induces collagen IV and fibronectin synthesis simulating ANG II roles (Zimpelmann and Burns 2009). This suggests that SPI exerts a protective effect in MCs attenuating fibrotic processes that may lead to renal injuries by reducing ANG (1‐7) expression in these cells.

MAS receptor expression was also decreased in the presence of aldosterone and SPI treatment as Figure 11 demonstrates. The MAS receptor is recognized as the receptor through which ANG (1‐7) activity is mediated (Santos et al. 2003) and, as with ACE2, both ANG (1‐7) and the MAS receptor exert contrasting effects depending on the tissue in question (Xu et al. 2017). Zimpelmann and Burns showed that MAS receptor antagonist decreases the growth stimulatory pathways induced by ANG (1‐7) in MCs, while AT1 and AT2 receptor antagonists were unable to reproduce these results (Zimpelmann and Burns 2009). On the other hand, ANG (1‐7) infusion accelerates kidney damage in diabetic rats by increasing expression of the AT1 receptor. Activation of this receptor in the proximal tubule induces an increase in blood pressure in response to intracellular ANG II, and reduces expression of the AT2 receptor, chronic activation of which has been found to reduce renal AT1 receptor function and blood pressure in obese rats. Infusion of ANG (1‐7) also decreases MAS receptor expression, which has been observed to be reduced in the kidneys of rats with chronic renal disease (Shao et al. 2008; Ali et al. 2013; Li and Zhuo 2013; Ng et al. 2014). However, low concentrations of ANG (1‐7) decrease the responsiveness of the AT1 receptor to ANG II, suggesting that ANG (1‐7) protects against the deleterious effects of ANG II (Clark et al. 2001). These results suggest that ANG (1‐7) has complex relationships with the AT1, AT2, and MAS receptors, and may potentiate or suppress the effects triggered by activation of different ANG receptors, depending on its concentration. Indeed, several recent publications have highlighted the crosstalk that occurs between the MAS and ANG II receptors (Karnik et al. 2017).

Despite the contrasting findings regarding the ACE2/ANG (1‐7)/MAS axis in the literature, our results using MCs demonstrate that aldosterone increases ACE activity in human mesangial cells, this activity was reduced when cells were previously treated with the antagonist of aldosterone receptor, suggesting that the aldosterone‐induced cell injuries through ANG II release were attenuated by pretreatment with spironolactone. The modulation of ACE activity suggested the contribution of aldosterone in renal tissue injury with the emphasis on the protective effect of mineralocorticoid antagonist. Moreover, spironolactone was also able to reduce ANG (1‐7) and MAS receptor levels which may lead to a beneficial effect on these cells since it seems that in MCs ANG (1‐7) simulates the role of ANG II. In conclusion our data show that both aldosterone and SPI can modulate both RAAS axes and therapeutic use of an aldosterone blocker can contribute in tissue for a reduction in glomerulusclerosis modulating the renin‐angiotensin system. We can suggest that the maintenance of ANG (1‐7) levels after aldosterone stimulation could be clinically relevant, indicating that the development of compounds that promote ANG II cleavage to increase ANG (1‐7) levels, yet await clinical trials to be applied to patients with renal disease.

## Conflict of Interest

None declared.
